# Feasibility of Online and On-Site Physical Activity Delivery Among Older Adults: Mixed Methods Pilot Randomized Controlled Trial

**DOI:** 10.2196/97824

**Published:** 2026-10-13

**Authors:** Anna Nilstomt, Johanna Gustavsson, Charlotte Bäccman, Linda Beckman, Finn Nilson, Erik Wästlund

**Affiliations:** 1 Service Research Centre (CTF) Karlstad University Karlstad Sweden; 2 Department of Social and Psychological Studies Karlstad University Karlstad Sweden; 3 Centre for Societal Risk Research (CSR) Karlstad University Karlstad Sweden; 4 Department of Political, Historical, Religious and Cultural Studies Karlstad University Karlstad Sweden; 5 Department of Public Health Karlstad University Karlstad Sweden

**Keywords:** physical activity, older adults, internet-based intervention, exercise adherence, attrition, mixed methods, Otago Exercise Program, pragmatic trial, digital health intervention, pilot study

## Abstract

**Background:**

Physical activity (PA) is central to healthy aging, yet interventions targeting older adults often report substantial attrition and variable adherence. Although digital and internet-based exercise programs broaden accessibility and flexibility, their ability to sustain engagement compared with traditional on-site formats remains unclear. Understanding how delivery mode influences attrition and adherence—and why participants persist or withdraw—is essential for designing inclusive and sustainable PA interventions.

**Objective:**

This pilot study evaluates the feasibility of delivering a 26-week PA intervention to older adults through 2 modes: online or on-site. Feasibility was assessed primarily by attrition, adherence, and participants’ experiences of factors influencing continued participation or withdrawal. Additionally, the study explores preliminary patterns in PA levels and physical and mental health outcomes to inform the design of a future definitive trial.

**Methods:**

This 26-week pragmatic pilot trial used an embedded convergent mixed methods design. Community-dwelling adults aged 60 years or older (N=35) from 3 residential areas in Sweden were cluster-randomized, without blinding, by site to an online (prerecorded video-based Otago exercises; n=12) or on-site (group-based, in-person Otago exercises; n=23) delivery mode. Feasibility outcomes included attrition and adherence. Exploratory outcomes included PA level (Godin-Shephard Leisure-Time Exercise Questionnaire), lower-body strength (Chair Stand Test), depressive symptoms (Geriatric Depression Scale), and health-related quality of life (EQ-5D-3L), assessed at baseline, midintervention (14 weeks), and postintervention (26 weeks). Exploratory quantitative analyses included t tests, chi-square tests, analysis of covariance, and mixed-design ANOVAs. We analyzed semistructured interviews with completers (n=17) and dropouts (n=7) using qualitative content analysis. Findings were integrated using the COM-B (Capability, Opportunity, Motivation-Behavior) framework.

**Results:**

The overall attrition rate was 37% (13/35), with no significant difference between delivery modes (*P*=.29). Most dropouts occurred within the first 2 weeks. Mean adherence appeared higher in the online delivery mode (mean 17.0, SD 6.7) than in the on-site delivery mode (mean 12.1, SD 10.1) but did not differ significantly (*P*=.15). Qualitative findings indicated that attrition was primarily driven by physical health issues and a perceived lack of fit, whereas adherence was supported by appeal and reward, routine and commitment, flexibility, feedback and PA overview, and (often) social presence. Mixed methods findings suggest that capability, opportunity, and motivation supported participation across both delivery modes. No intervention-related adverse events were reported.

**Conclusions:**

Both online and on-site delivery modes for PA interventions appeared feasible for many older adults. Although no statistically significant between-group differences were observed, the study was not powered to evaluate effectiveness. Feasibility may depend more on supporting capability, opportunity, and motivation than on delivery mode. Larger, adequately powered trials are needed to test effectiveness, identify optimal formats for different populations, and evaluate strategies to reduce early attrition.

**Trial Registration:**

ClinicalTrials.gov NCT05885308; https://clinicaltrials.gov/ct2/show/NCT05885308

## Introduction

Physical activity (PA) promotes healthy aging, increases longevity, and reduces disease burden; even small increases have been shown to improve longevity and reduce disease risk [1,2]. Thus, studying attrition and adherence can provide valuable insights into PA participation (ie, PA engagement) and improve the design of future PA programs (ie, structured exercise plans). The terms attrition and adherence differ and highlight different aspects: attrition refers to participants withdrawing or being lost to follow-up prematurely (ie, dropouts) [3-5], whereas adherence is the degree to which a participant follows the intervention’s recommendations [6]. A review by Picorelli et al [7] has shown that older people’s exercise programs have adherence rates of 58% to 77% and dropout rates of 14% to 35%. Other studies have reported higher attrition rates of 36% [8] and 43% [9], with most attrition occurring within the first 3 months [8,10]. Although attrition is straightforward to assess, adherence is assessed using various methods, such as self-report diaries and pedometers [6]. However, although adherence and attrition are quantitative measures that provide valuable information about an intervention and help interpret its findings [3,4,11], understanding the reasons behind these numbers is central to improving PA programs and interventions [6,12].

Prior research has identified several person-related factors associated with PA attrition and adherence among older adults [7,8,10,13], such as disease burden and physical and mental health [7,13-15], competence and self-efficacy [10,13], and socioeconomic status (SES) [7,10]. For example, being overweight or lonely, having low PA engagement [10], losing interest [12], or not feeling that the training meets one’s needs [16] are associated with attrition, whereas finding the training enjoyable, beneficial, or goal-aligned is associated with adherence [13]. Besides individual determinants, aspects related to the PA program also matter for older adults’ PA engagement. For example, program duration affects both dropout and adherence [13,17], while the setting (ie, facility- or home-based) predicts dropout, and the format (ie, group- or individual-based) impacts adherence [17]. Supervision by exercise professionals can also improve adherence [7,13]. Additionally, PA education that sets realistic expectations, teaches health benefits, PA that promotes positive social interaction which fosters a sense of belonging and support, and self-monitoring tools that offer clear, objective, user-friendly data can enhance older adults’ PA adherence [13].

Factors linked to older adults’ PA engagement can be understood through the COM-B (Capability, Opportunity, Motivation-Behavior) model, which broadly categorizes behavioral influences by assuming that each behavior is influenced by capability, opportunity, and motivation [18,19]. Capability (physical or psychological abilities) and motivation (automatic or reflective motivation) relate to intrinsic factors, whereas opportunity (social or contextual conditions) pertains to external factors. Each component directly impacts behavior, while opportunity and capability can indirectly impact behavior through motivation [18]. A review by Yang et al [20] found that all COM-B components can influence older adults’ PA adherence and endorsed its use in designing and assessing PA interventions for this group. Yang et al [20] also emphasized the need to examine how internet-based interventions (ie, interventions delivered via digital means) influence PA engagement among older adults. This highlights the potential usefulness of the COM-B model in designing internet-based interventions to increase PA among older adults.

While digitally delivered PA interventions are generally considered feasible and acceptable for many older adults, engagement may be influenced by digital literacy, perceived relevance, safety, intervention quality, and access to social support [21]. Consequently, their usefulness in real-life settings may depend not only on access to technology but also on how well the intervention accommodates participants’ capabilities, preferences, and everyday circumstances [13,21,22]. Recent evidence suggests that synchronously supervised remote exercise may produce physical-function outcomes comparable to those of equivalent in-person exercise among older adults with prefrailty [23]. However, this evidence primarily concerns live, professionally supervised exercise and may not generalize to low-support asynchronous interventions delivered through prerecorded videos. Evidence also remains limited regarding long-term engagement and feasibility in such formats [23-25]. Furthermore, additional comparisons of long-term effectiveness between internet-based, face-to-face, and paper-based interventions are needed to inform implementation in community settings [24,25]. Prior research suggests that combining print- and web-based delivery modes may minimize attrition, as internet-based interventions have been associated with higher dropout rates among older-aged participants [26]. Given these uncertainties, pragmatic feasibility designs [27-30] are warranted to determine whether intervention and study procedures can be implemented under realistic conditions. Before assessing comparative effectiveness, it is important to establish the feasibility of key study processes, including adherence, attrition, acceptability, and outcome measurement, to ensure suitability for large-scale implementation [28,30]. Moreover, a better understanding of adherence [6] and withdrawal [12] may help improve intervention delivery and inform the design of future definitive trials.

This study aimed to evaluate the feasibility of delivering a 26-week PA intervention to older adults through 2 delivery modes: online (ie, PA sessions accessible via YouTube [Google LLC/Alphabet Inc]) or on-site (ie, PA sessions at the community host’s facility in a group setting). Feasibility was assessed primarily in terms of attrition, adherence, and participants’ experiences of factors influencing continued participation or withdrawal. An additional aim was to explore preliminary patterns in PA levels and physical and mental health outcomes to inform the design of a future definitive trial. The aims were operationalized into the following quantitative, qualitative, and mixed methods research questions in accordance with the mixed methods procedure outlined by Creswell and Clark [31]:

- What patterns of attrition, adherence, PA levels, and physical and mental health outcomes were observed among participants receiving the intervention through online or on-site delivery modes? (quantitative)
- What were the reasons for attrition and the motivations for adherence during the intervention? (qualitative)
- How do the qualitative findings, interpreted through the COM-B framework, help explain the quantitative patterns of attrition and adherence? (mixed methods)

## Methods

### Participants

The quantitative sample included 35 older adults (27 women) who lived independently in municipality-owned apartments across 3 residential areas (k=3) in a midsized town in Sweden. Each residential area included community hosts who cleaned and maintained common areas while organizing day-to-day activities for residents.

The median age ranged from 76 to 80 years, and the participants had an average BMI of 27.3 (SD 3.75) kg/m^2^. Most participants lived alone (n=28) and had access to a smartphone, computer, or tablet with Wi-Fi (n=31). Half of the participants (n=17) used some form of assistive technology for aging populations. Of the 35 participants, 23 from 2 residential areas were allocated to the on-site delivery mode, and 12 from the third residential area were allocated to the online delivery mode.

The participants self-rated their SES as above average (mean 6.46, SD 1.85; range 1-10). Their self-reported health status was high (mean 0.87, SD 0.11; range 0.3402-0.9694), and, according to the Godin-Shephard Leisure-Time Exercise Questionnaire (GSLTEQ), the majority (n=21) were sufficiently active, while only 9 were insufficiently active. The overall degree of depressive symptoms was low (mean 4.17, SD 3.85; range 0-20), as measured by a 20-item yes-or-no screening tool. However, 10 participants reported 6 or more depressive symptoms, potentially indicating depression [32].

There was a difference between the online and on-site delivery modes in cohabitation status, with participants in the online delivery mode more likely to be living with a partner than those in the on-site delivery mode (*χ*^2^_1_=5.21, *P*=.02, Bayes factor of H_0_ relative to H_1_ [BF_01_]=3.88). The expected count was less than 5; however, prior research indicates that this statistic is robust to small expected cell frequencies [33,34]. There were no other statistically significant differences with respect to age (*P*=.57), SES (*P*=.30), or GSLTEQ (*P*=.37; all 3 were nonsignificant). See Table 1 for a detailed description of the sample by online and on-site delivery mode.

Of the total sample (N=35), 24 participants were interviewed (7 dropouts and 17 completers). See Table 2 for a description of the sample included in the qualitative dataset.

**Table 1:** Sample description of participants in the online and on-site delivery modes (N=35).

Baseline characteristics		Delivery mode	
		Online (n=12)	On-site (n=23)
Age (years)^a^, median		76-80	81-85
Gender, n			
	Female	9	18
	Male	3	5
Cohabitation status, n			
	Living alone	7	21
	Living with a partner	5	2
Using aids, n			
	Yes	5	11
	No	7	11
Have a smartphone/computer/tablet with Wi-Fi, n			
	Yes	11	20
	No	0	3
Subjective socioeconomic status^b^, mean (SD)		6.92 (1.17)	6.22 (2.11)
BMI (kg/m^2^), mean (SD)		26.90 (3.56)	27.60 (3.93)
Godin-Shephard Leisure-Time Exercise Questionnaire^c^, mean (SD)		45.10 (27.6)	36.70 (25.40)
	Sufficiently active, n	8	13
	Moderately active, n	2	3
	Insufficiently active, n	2	7
EQ-5D-3L^d^, mean (SD)		0.90 (0.070)	0.86 (0.12)
The Geriatric Depression Scale index^e^, mean (SD)		4.17 (3.13)	4.17 (4.24)
	≥6, n	4	6
	<6, n	8	17

^a^Age was measured as a categorical variable (range 60-64 to 105 years and older).

^b^Subjective socioeconomic status index range 1-10.

^c^Godin-Shephard Leisure-Time Exercise Questionnaire index range 0-119.

^d^EQ-5D-3L index range 0.3402-0.9694.

^e^Geriatric Depression Scale index range 0-20.

**Table 2:** Sample description of participants in the qualitative dataset^a^.

Characteristics			Dropped out		Completed
Delivery mode, n					
	Online	2		9	
	On-site	5		8	
Age (years)^b^, median			81-85		76-80
Gender, n					
	Female	5		14	
	Male	2		3	
Cohabitation status, n					
	Living alone	6		11	
	Living with a partner	1		6	

^a^The total sample size was 24; of these, 7 interviewees dropped out of the intervention, and 17 completed the intervention.

^b^Age was measured as a categorical variable (range 60-64 to 105 years and older).

### Study Design

#### PA Intervention and Delivery Modes

PA was defined as either exercise sessions according to the Otago Exercise Program (OEP), which focuses on strengthening and balance exercises and includes elements of stretching and walking [35,36], or any exercise program chosen by the participants, such as a popular TV-broadcast training session for older adults. The OEP is an effective health promotion program for older adults that can reduce falls [37,38] and improve strength, balance, and health status [38-42]. Two delivery modes—online or on-site —were used in this pilot trial to assess the feasibility and acceptability of a 26-week PA intervention among older adults. Delivery mode was theoretically informed by the COM-B model [18,19]. The on-site delivery mode was designed to target social opportunity and was therefore group-based, open to all area residents, and held at the residential common facilities. A community host led two 45-minute OEP sessions per week (ie, a total of 52 sessions), using background guidance from the 37-minute OEP instruction film on YouTube.

The online delivery mode targeted physical opportunity, and participants were given prerecorded, asynchronous OEP sessions on YouTube. The videos included a 37-minute complete workout and 3 videos focusing on warm-up, strength, or balance. This format allowed participants the flexibility to exercise at their convenience. No live, instructor-led online sessions or group meetings were provided as part of the intervention; participants exercised independently. Instructions for accessing the videos were provided, and community hosts assisted upon request if participants experienced difficulties accessing them. No additional instructions or interactive elements were provided.

Data were collected using an embedded concurrent mixed methods design, with interviews embedded within the intervention [31]. Thus, the sample of interviewees was nested within the total study sample. A pragmatic pilot-trial approach was chosen to evaluate the feasibility [28-30] of a future pragmatic trial intended to examine the effectiveness of the intervention in a natural setting [43,44]. See Multimedia Appendix 1 for the CONSORT (Consolidated Standards of Reporting Trials) checklist.

#### Quantitative Data

Self-reported data were collected at 3 time points: baseline (T0), midintervention (T1), and postintervention (T2). T1 and T2 were conducted 14 and 26 weeks after the start of the intervention, respectively.

#### Qualitative Data

Participants were interviewed either when they dropped out or completed the study. The interviews aimed to examine participants’ experiences with the intervention, thereby enriching the overall understanding of the intervention [45]. Within this design, the quantitative analysis was prioritized and served as the primary focus, while the qualitative analysis played a complementary role [46].

### Procedure

All residents in the 3 areas who had a disability pension or were aged 65 years or older were eligible to participate. In April 2022, 700 individuals in the 3 residential areas were approached by the research team through local advertisements (ie, posters in stairwells and letters in residents’ mailboxes) and information meetings at the community hosts’ facilities. Both written and oral information about the study was provided to potential participants. Of those approached, 35 provided informed consent to participate and enrolled in the study. The recruited sample was randomized, without blinding, by residential area, using a lottery, to the delivery mode: 2 residential areas were randomized to the on-site mode (n=23), and 1 residential area to the online mode (n=12). Given that randomization was based on the 3 residential areas included in the study, it should be noted that delivery mode was confounded by area-specific contextual factors. Consequently, the analyses should be interpreted as exploratory only. As this was a pilot study, a sample size calculation was not required [28,30]. However, for a future randomized controlled trial, the sample size was precalculated at 128 participants, with 64 in each condition, using G*Power 3 [47] with a medium effect size of 0.5 [48], a 95% 2-sided confidence level, and 80% statistical power. This indicates a recruitment shortfall in this pilot study and limits its statistical power and generalizability; comparisons between delivery modes should therefore be considered exploratory.

All participants completed the T0 assessment 0-14 days before the start of the intervention. At the start of the intervention in May 2022, all participants received a Swedish-language paper manual (see Gawler [35]) and a PA lecture that included a demonstration of the OEP. During the PA demonstration, participants were notified by the research team whether they had been allocated to the online or on-site delivery mode and were informed about the intervention goal (ie, 2 walks and OEP or another exercise twice weekly). All participants were asked to register PA engagement in an activity calendar for the duration of the study. The activity calendars were collected at T2 or when participants quit the study.

During the first 2 weeks following the start of the intervention, participants were offered 2 question-and-answer sessions led by the research team at the shared community facilities. The researchers were available by phone for questions throughout the study and met with participants at the 2 subsequent assessment points (ie, T1 and T2).

Interviews were conducted at T2 with participants who completed the intervention (n=17) or dropped out (n=7) consecutively between fall 2022 and early 2023. Interviews with dropouts focused on their reasons for withdrawal, whereas interviews with completers focused on how the intervention components (eg, delivery mode, pedometer, and activity calendar) influenced their experiences. Both interview guides included questions about the participants’ overall experience with the intervention.

The interviews took place either in the participant’s home or in a private area of the community hosts’ office, based on the participant’s preference, and lasted an average of 60 minutes. All interviews were audio-recorded and transcribed verbatim. The interviewers were the first (AN) and second (JG) authors (n=16 and n=8, respectively). Interviewers’ preunderstanding was managed through self-reflective notes.

The study was retrospectively registered at ClinicalTrials.gov under the registration number NCT05885308 on May 30, 2023. Participant enrollment began in April 2022.

### Measures

The baseline questionnaire assessed demographic information such as gender, cohabitation, BMI, subjective SES, use of assistive technology, and access to technology (smartphones, computers, and tablets). Age was categorized into 4-year intervals, ranging from “60-64” to “105 years and older.”

### Primary Feasibility Outcomes

#### Adherence

Any day with registered steps using the Rubicson pedometer indicated a walk. Participants were instructed to always wear the pedometer and report the number of steps each day in an activity calendar. Adherence was defined as any week with two 30-minute walks and 2 registered exercise sessions. Adherence scores were calculated as the sum of all weeks with two 30-minute walks and 2 training sessions, and thus ranged from 0 to 26.

#### Attrition

Attrition was defined as the number of participants who dropped out (n), and the attrition rate was the percentage of participants who dropped out (%).

### Secondary Exploratory Outcomes

#### Depression

Depressive symptoms were assessed using the Swedish Geriatric Depression Scale (GDS; [32]). This 20-item yes-or-no screening tool scores from 0 to 20, with higher scores indicating more symptoms. A score of 6 or more suggests possible depression, whereas a score of 5 or lower suggests otherwise [32]. Cronbach α was 0.82.

#### Health-Related Quality of Life

Health-related quality of life (EQ-5D-3L; [49]) was self-rated across 5 dimensions (mobility, self-care, usual activities, pain/discomfort, and anxiety/depression) at 3 severity levels (no, moderate, or severe problems). Index values, based on the Swedish value set [50], ranged from 0.3402 to 0.9694, indicating the lowest to highest health-related quality of life, respectively. The interitem correlation was 0.39.

#### PA Level

The GSLTEQ [51] assessed weekly PA using 4 items. Participants self-reported the frequency of mild, moderate, and strenuous PA for at least 15 minutes per week on an 8-point scale (0-7 days). The PA scale index ranged from 0 to 119, with higher scores indicating higher PA levels. The total score, calculated using Godin’s [51] standard procedure, classified individuals as “sufficiently,” “moderately,” or “insufficiently active.” The interitem correlation (Pearson correlation, *r*) was 0.4, which falls within the recommended range of 0.2-0.4 [NEW REFS 52, 53].

#### Physical Capacity Test

The chair stand test assessed lower-body strength, with a higher number of completed stands indicating greater strength. The standard procedure described by Rikli and Jones [54] was used.

### Data Analysis

#### Quantitative Analyses

Statistical analysis was conducted using SPSS version 29 (IBM Corp) with an α level of .05. Descriptive statistics were used to describe the baseline (T0) characteristics of all participants. Missing data were classified as group-level dropouts (ie, attrition) when missingness was due to participants not continuing the pilot intervention, that is, when data from T0, T1, or T2 were missing, or as individual-level inactivity. Participants were not treated as dropouts if single-item values were missing randomly; in these few cases, analyses were based on available values. Thus, no missing data were imputed. Data recorded in the activity calendar before the intervention started were excluded to ensure consistency. Outliers were identified using *z* scores.

Group differences were assessed using independent-samples 2-sided *t* tests, Mann-Whitney *U* tests, and a chi-square test for proportions [33,34,55]. Cohabitation (dichotomous) was identified as a significant (*P*=.02) baseline difference between the online and on-site delivery modes. An analysis of covariance, with cohabitation as a covariate, assessing total adherence to the intervention goal across delivery modes showed no significant difference (*P*=.41) and was therefore not considered in further analyses.

Adherence was assessed using an analysis of covariance, with delivery mode (ie, online or on-site ) as the independent variable, cohabitation as a dichotomous covariate, and total adherence to the intervention goal as the dependent variable. Weekly adherence was inspected graphically.

Exploratory analyses of PA levels and physical and mental health were conducted to generate preliminary estimates and identify patterns, using 4 mixed-design ANOVAs, with delivery mode (online or on-site) as the independent variable and scores from the GSLTEQ, chair stand test, GDS, and EQ-5D-3L across T0-T2 as dependent variables.

#### Qualitative Analyses

Interviews from dropouts and completers were analyzed and presented separately to address the research questions concerning attrition and adherence, respectively. The analysis was conducted in NVivo 14 (Lumivero). All interview data were analyzed using content analysis according to Graneheim and Lundman [56]: first, the transcripts were read in their entirety multiple times; second, meaning units were identified and coded; and third, categories emerged from the codes using abductive reasoning. The first author (AN) completed the coding, and the categories were discussed among the authors. Quotes were selected to exemplify empirical validity.

#### Mixed Methods Analysis

Before integrating the quantitative and qualitative data, each component was collected and analyzed separately according to its methodological quality standards to ensure methodological rigor and trustworthiness [31]. The research team then discussed how the qualitative findings augmented the understanding of the quantitative results in relation to intervention feasibility. Specifically, interview data from dropouts and completers were combined with the trial results on attrition and adherence and interpreted through the COM-B framework [18,19]. The mixed methods results are presented narratively and in a joint display.

### Ethical Considerations

The study was approved by the Swedish Research Ethics Committee (approval number Dnr 2021-05133). Participation was based on informed consent, whereby participation was voluntary, and participants had the right to discontinue participation or refrain from answering questions at any point. Participants received no financial compensation. All data were pseudonymized, and all identifying information was stored separately. Quantitative data were aggregated and analyzed at the group level to ensure participants’ privacy. For qualitative data, quotes illustrating the emerging categories were pseudonymized and selected carefully to avoid infringing on participants’ integrity or risking identification.

### Researchers’ Description

The first (AN) and second (JG) authors conducted the interviews (n=16 and n=8, respectively). Our preunderstanding was managed through self-reflective notes. The research team has substantial experience in quantitative and qualitative research involving older adults and behavior change. Its members have backgrounds in psychology, nursing, public health, and physical therapy.

## Results

### Quantitative Findings

#### Overview

The quantitative findings are presented below, structured by feasibility and exploratory outcomes.

#### Feasibility Outcomes

##### Attrition

A total of 22 participants completed the program, resulting in a 37% (13/35) attrition rate (Figure 1). Most attrition occurred during the first 2 weeks of the intervention. Three participants dropped out from the online delivery mode, and 10 dropped out from the on-site delivery mode. Although the number of dropouts appeared lower in the online group, the difference was not statistically significant (χ^2^_1_=1.120, *P*=.29, 95% CI –14.899 to 43.952). Baseline characteristics did not differ significantly between completers and dropouts with respect to age (*P*=.78), gender (*P*=.40), cohabitation status (*P*=.17), use of aids (*P*=.54), access to a smartphone, computer, or tablet with Wi-Fi (*P*=.30), subjective socioeconomic status (*P*=.13), BMI (*P*=.52), GSLTEQ (*P*=.15), EQ-5D-3L (*P*=.06), or GDS (*P*=.22). Baseline characteristics of all participants are presented in Table 3.

**Figure 1:**
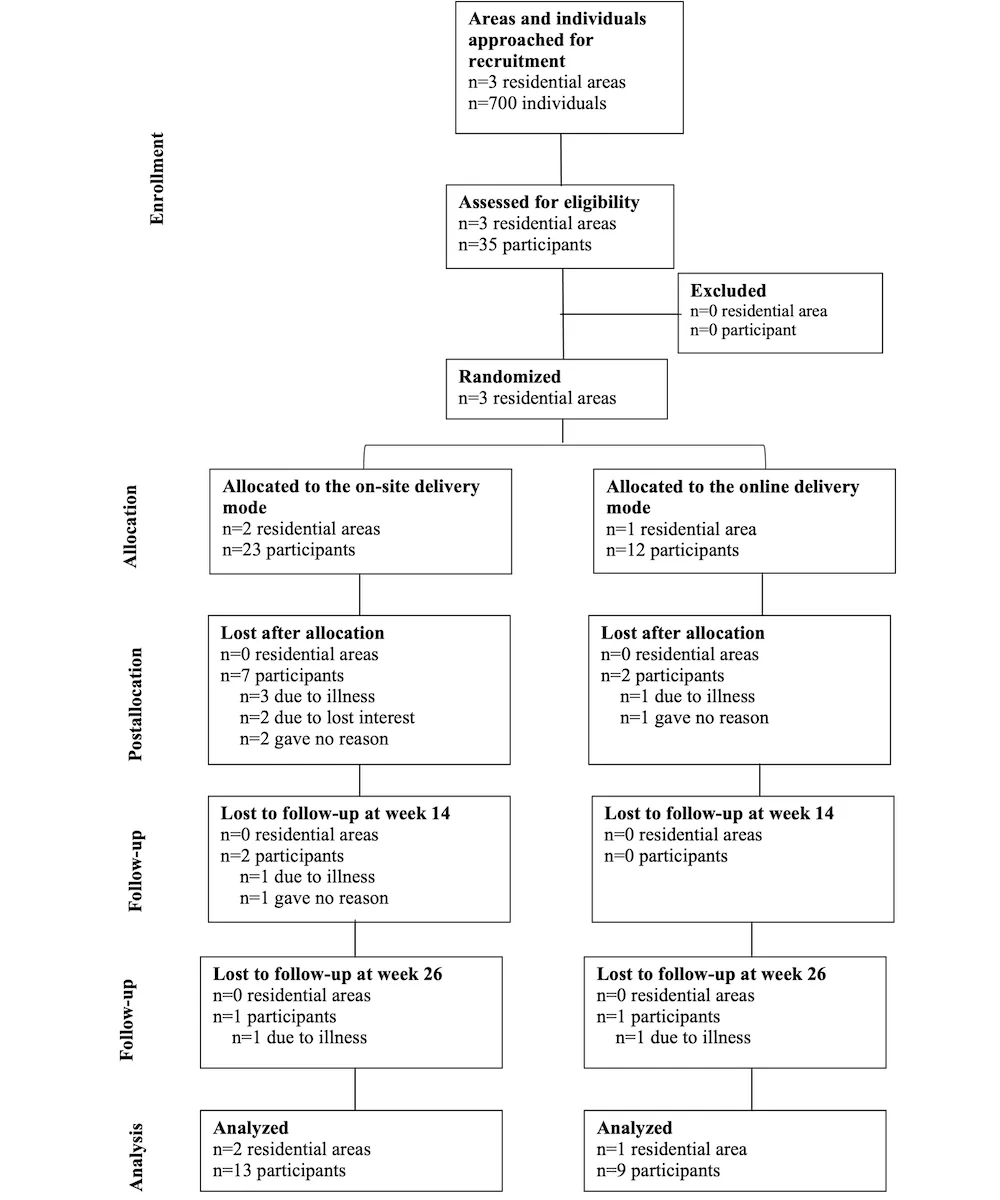
CONSORT (Consolidated Standards of Reporting Trials) participant flow diagram.

**Table 3:** Baseline characteristics of all participants, stratified by intervention completion status (dropouts vs completers; N=35).

Characteristics			Dropped out (n=13)		Completed (n=22)
Age (years)^a^, median			76-80		76-85
Gender, n					
	Male	9		18	
	Female	4		4	
Cohabitation status, n					
	Living alone	12		16	
	Living with partner	1		6	
Using aids, n					
	Yes	7		9	
	No	6		12	
Have a smartphone/computer/tablet with Wi-Fi, n					
	Yes	11		20	
	No	2		1	
Subjective socioeconomic status^b^, mean (SD)			7.08 (0.57)		6.09 (0.35)
BMI (kg/m^2^), mean (SD)			27.90 (3.99)		27.00 (3.68)
Godin-Shephard Leisure-Time Exercise Questionnaire^c^, mean (SD)			31.20 (5.98)		44.60 (5.90)
	Sufficiently active, n	7		14	
	Moderately active, n	2		3	
	Insufficiently active, n	4		5	
EQ-5D-3L^d^, mean (SD)			0.82 (0.14)		0.91 (0.07)
5-item Geriatric Depression Scale^e^, mean (SD)			5.23 (4.42)		3.55 (3.42)
	Score >6, n	6		4	
	Score <6, n	7		18	

^a^Age was measured as a categorical variable (range 60-64 to 105 years and older).

^b^Subjective socioeconomic status index range 1-10.

^c^Godin-Shephard Leisure-Time Exercise Questionnaire index range 0-119.

^d^Health-related quality of life index range 0.3402-0.9694.

^e^Geriatric Depression Scale index range 0-20.

#### Adherence to the Intervention Goal

Participants in both delivery modes demonstrated engagement with the intervention throughout the 26-week period. Weekly adherence to the intervention goal of completing 2 walks and 2 exercise sessions (ie, OEP or other workouts) was inspected graphically and was higher in the online delivery mode than in the on-site delivery mode (Figure 2). Adherence in the online delivery mode peaked in weeks 3, 14, and 19, with complete adherence occurring only in week 3, whereas the on-site delivery mode showed steadier adherence throughout the intervention. The average adherence rates were 65.4% and 46.6%, respectively.

However, an exploratory between-participant comparison revealed no significant difference (*t*_24_=–1.477, *P*=.15, 95% CI –2.60 to 12.35) between the online mode (mean 17.00, SD 6.70, n=10) and the on-site mode (mean 12.10, SD 10.10, n=16).

**Figure 2:**
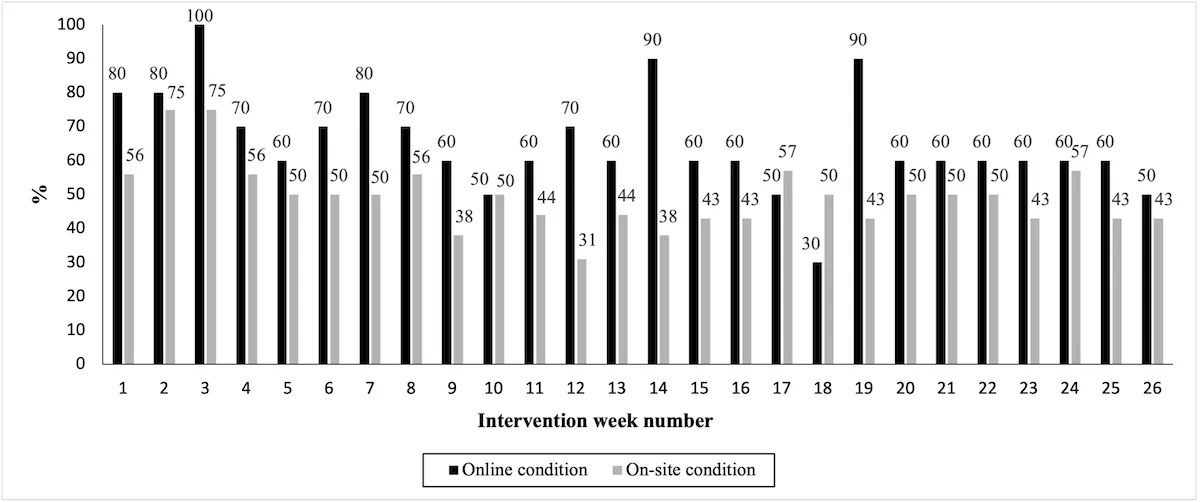
Weekly proportion of adherence to the intervention goal.

### Exploratory Outcomes

PA levels and physical and mental health outcomes were assessed to provide preliminary estimates for a future definitive trial. Results should be interpreted cautiously. Exploratory analyses did not indicate a significant main effect of delivery mode on total PA level, as assessed by the GSLTEQ (*F*_1,18_=0.43, *P*=.84, η_p_^2^=0.002). There was no significant interaction between delivery mode and time on GSLTEQ scores (*F*_2,36_=0.74, *P*=.48, η_p_^2^=0.039). There was a main effect of time on GSLTEQ scores (*F*_2,36_=4.46, *P*=.02, η_p_^2^=0.20), with both delivery modes showing an increase in GSLTEQ scores over time. Post hoc tests using Bonferroni-corrected pairwise comparisons revealed that GSLTEQ scores were significantly higher at midintervention than at preintervention (*P*=.03). GSLTEQ scores did not differ significantly at postintervention (*P*=.28) compared with preintervention or midintervention (*P*=.58). Descriptive statistics are presented in Table 4.

Exploratory analyses did not reveal a significant main effect of delivery mode on physical strength (ie, chair stand test; *F*_1,16_=1.38, *P*=.26, η_p_^2^=0.079). There was a main effect of time on chair stand scores (*F*_2,32_=13.43, *P*<.001, η_p_^2^=0.46), with both delivery modes showing increases in chair stand scores across the 3 time points. Post hoc tests using Bonferroni-corrected pairwise comparisons revealed that chair stand scores were significantly higher at both midintervention and postintervention than at preintervention (*P*<.001 for both comparisons). There was no significant difference between midintervention and postintervention scores (*P*>.99). There was no significant interaction between delivery mode and time (*F*_2,32_=0.67, *P*=.52, η_p_^2^=0.04). Exploratory analyses revealed no significant effects for either the GDS or EQ-5D-3L. For the GDS, there was no significant interaction between delivery mode and time (*F*_2,36_=0.88, *P*=.42, η_p_^2^=0.047), no significant main effect of time (*F*_2,36_=1.07, *P*=.35, η_p_^2^=0.056), and no significant main effect of delivery mode (*F*_1,18_=0.06, *P*=.94, η_p_^2^=.000). Similarly, for the EQ-5D-3L, there was no significant interaction between delivery mode and time (*F*_2,32_=0.83, *P*=.45, η_p_^2^=0.049), no significant main effect of time (*F*_2,32_=1.28, *P*=.29, η_p_^2^=0.074), and no significant main effect of delivery mode (*F*_1,16_=1.99, *P*=.18, η_p_^2^=.11). Owing to the pilot design and limited sample size, these findings should be interpreted cautiously.

**Table 4:** Descriptives for each delivery mode (online or on-site) and mixed ANOVA statistics for study variables (n=22).

Measure		Delivery mode					Test statistics			
		Online (n=9)		On-site (n=13)		ANOVA				
		n	Mean (SD)	n	Mean (SD)	Effect		*F* ratio	*df*	η_p_^2^
Godin-Shephard Leisure-Time Exercise Questionnaire^a^										
	Time 1	8	40.80 (29.20)	12	44.00 (27.80)	Group		0.043	1, 18	0.002
	Time 2	8	57.80 (26.40)	12	54.20 (31.40)	Time		4.460^b^	2, 36	0.200
	Time 3	8	45.60 (19.70)	12	53.00 (26.10)	Group × time		0.740	2, 36	0.039
Chair stand										
	Time 1	8	9.25 (3.73)	10	11.80 (3.19)	Group		1.38	1, 16	0.079
	Time 2	8	12.10 (5.30)	10	13.40 (2.68)	Time		13.43^c^	2, 32	0.460
	Time 3	8	12.30 (3.33)	10	14.10 (3.51)	Group × time		0.67	2, 32	0.040
Geriatric Depression Scale^d^										
	Time 1	8	3.38 (2.67)	12	4.00 (4.05)	Group		0.006	1, 18	0.000
	Time 2	8	3.00 (2.62)	12	3.17 (2.59)	Time		1.07	2, 36	0.056
	Time 3	8	3.50 (2.51)	12	3.00 (2.73)	Group × time		0.88	2, 36	0.047
EQ-5D-3L^e^										
	Time 1	7	0.94 (0.034)	11	0.89 (0.087)	Group		1.99	1, 16	0.110
	Time 2	7	0.92 (0.059)	11	0.87 (0.093)	Time		1.28	2, 32	0.074
	Time 3	7	0.93 (0.037)	11	0.86 (0.092)	Group × time		0.83	2, 32	0.049

^a^Godin-Shephard Leisure-Time Exercise Questionnaire index range 0-119.

^b^*P*<.05.

^c^*P*<.001.

^d^Geriatric Depression Scale index range 0-20.

^e^Health-related quality of life index range 0.3402-0.9694.

## Qualitative Findings

### What Are the Participation Reasons for Attrition?

#### Overview

Follow-up interviews with participants who dropped out helped us understand their reasons for attrition. Two categories emerged from the qualitative analysis: *physical issues* and *it is not for me.*

#### Physical Issues

Several participants could not complete the intervention due to physical issues, primarily pain and fatigue.

When I signed up for the study, I felt so healthy and energetic. I thought this would be great as it gave me a boost [to be active]. Then I became so ill that I was hospitalized.

Participant 4

These symptoms were sometimes related to chronic conditions, such as back and leg pain, with or without relapses, or injuries associated with, for example, a stroke. A couple of participants had acute viral or other infections that required hospitalization, preventing their participation. Those who dropped out due to physical issues initially hoped to participate and often felt that they had overestimated their abilities.

I really want to, but I can’t right now.

Participant 1

Some expressed sadness about this realization.

#### It Is Not for Me

Other participants withdrew because they felt out of place, either finding themselves too active or too fragile. Those who believed they were too active and healthy compared with the other participants did not feel old enough for the intervention and suggested age-segmented PA groups to foster a greater sense of common ground among participants.

I’m insanely much more mobile and energetic [than the other participants] and can still give myself daily doses of physical activity if I want to.

Participant 2

Participants who felt they did not belong also reported valuing the freedom to choose how to spend their time and not wanting to adhere to a specific program. Consequently, they expressed no interest in committing to regular participation in this type of PA.

### What Are the Participants’ Motivations for Adherence?

#### Overview

Follow-up interviews with intervention completers showed that study participation motivated PA engagement, especially at the start. However, several other motivators for PA were also identified. This analysis focuses on those motivators that can be utilized to develop interventions. Five categories emerged from the analysis: appeal and reward, routine and commitment, flexibility, feedback and PA overview, and presence of others.

#### Appeal and Reward

The participants’ motivation for training was driven by its appeal and rewards, which were linked to its meaningfulness, convenience, and satisfaction. When participants valued the activity, understood its purpose, and found it both easy and valuable, its appeal and their motivation to engage increased. Conversely, when these aspects were lacking, their motivation decreased. Experiencing health benefits such as increased energy, improved balance, and enhanced well-being reinforced PA engagement. Enjoyment and mastery of the activity further motivated PA.

I’ve experienced it as good. And every time you go to this training, you long to go back...I think it feels great to know that I can handle it. I’m over 80 years old, so after all...it’s not so bad that I keep up with it. Many times, I’m proud of myself.

Participant 11

Additionally, feeling safe and comfortable was a prerequisite for a positive and appealing PA experience. Over time, some participants’ initial perceptions of the OEP’s appeal improved as they observed positive health effects. Meanwhile, others, despite experiencing a few immediate benefits from the OEP, were motivated by the anticipation of future rewards.

I may not need it [this training] right now, but I still need it right now to help me in the future.

Participant 6

#### Routine and Commitment

Routine and commitment involved establishing structure and habits, which boosted motivation for PA. Consciously choosing to participate in PA increased motivation, as it helped reduce scheduling conflicts during designated PA time.

Well, you started little by little, and suddenly you realized that you were out walking every single day. At first, you couldn’t do much; your cardio fitness hindered you, but in the end, you didn’t think about what you were doing.

Participant 6

This deliberate choice also facilitated sustained PA engagement if, for example, illness temporarily disrupted their PA habits. Over time, some participants gained a newfound understanding of how regular PA enhanced their well-being. Engaging in PA led some participants to develop automatic PA habits, encouraging continued training even after the intervention ended. As participant 7 noted, "...it has become a bit of a structure. Even though it's not ongoing anymore...it still made you do something every week. We continue with [exercise on] Thursdays."

#### Flexibility

Flexibility, which included options for workout days, locations, materials, equipment, and levels, was central to motivation across both intervention groups. For instance, some participants happily used the provided pedometers, whereas others found them discouraging and preferred their own activity trackers or apps. Flexibility in exercise tasks also appeared to be motivating, as participants found repeating the same exercises for an extended period monotonous and demotivating, prompting a desire to change their routines.

Different preferences for flexibility were noted between the two intervention groups. The on-site participants enjoyed attending 2 sessions each week and having the option to exercise at home if they could not attend a session. The online participants appreciated the option to choose between workout media (ie, the paper manual and YouTube), although many preferred the paper manual to the video. Reasons for this preference varied; some had difficulty accessing the video, whereas others lacked an appropriate setting at home. Some found the video redundant after a few viewings.

I think [the YouTube video] was a good introduction...You watched it in the beginning, and then you didn't need to watch it every time. No, instead, that folder was better.

Participant 8

#### Feedback and PA Overview

Feedback and PA overview involved gathering input and summarizing activities that support and motivate PA. Participants enjoyed and benefited from tracking their PA with the activity calendar, which enabled them to reflect on and compare their activity levels over time. However, the lack of personal feedback on PA execution and expert consultation regarding physical issues during exercise led to some insecurity and limited PA participation. Some participants set individual goals and felt motivated by pride and a sense of achievement when tracking their activity.

I see that I can, I’m capable of walking.

Participant 9

Others felt guilty when noticing low activity levels, which spurred them to be more active. However, self-disappointment and performance anxiety generally had a demotivating effect on PA. Some participants lost interest in the activity calendar after recognizing their workout patterns, but many still valued it and wished to continue using it. During the postassessment, the researchers collected the activity calendars for data extraction, and several participants requested their activity calendars back. A couple of participants noted that the activity calendar served as a memory cue, and without it, they often forgot to exercise.

A couple of participants noted that the activity calendar served as a memory cue, and without it, they often forgot to exercise. And now I have to admit that – and it’s almost been a disadvantage – that this calendar has disappeared. Because now I forget to do it.

Participant 6

#### Presence of Others

The presence of others refers to people or animals in one’s environment who can encourage and inspire PA. Most participants, regardless of whether they participated in the online or on-site delivery mode, valued this presence during exercise. The on-site participants often appreciated the social element, which fostered a sense of community, belonging, and obligation.

And there’s also this thing where you think, “Well...wonder why he or she isn’t here today?” Or you get asked, “So, where were you last Thursday or Tuesday?” It creates a certain consistency.

Participant 13

They valued interactions, especially those living alone, but wished for more participants to enhance group dynamics and energy. Online participants also found the presence of others important for their PA. They often took the initiative to exercise with a partner, go to the gym, or take walks with friends and neighbors, combining exercise with social interaction.

One has to connect with someone; one doesn’t feel so strong alone. I know, it’s easy to give up [with the training] when you are alone.

Participant 10

However, not all participants in the study found social interaction motivating. Some preferred exercising without social commitment, indicating that not everyone is motivated by social interaction. For example, while some online participants desired future group PA, others preferred to work out alone and neither felt that others influenced their PA engagement.

## Mixed Methods Findings

From a COM-B perspective (see Table 5), capability, opportunity, and motivation appeared to shape participation across both delivery modes. Early attrition was primarily related to reduced capability due to illness and health limitations, whereas adherence was supported by social support, shared intervention components, self-monitoring, flexibility, enjoyment, and habit formation. Together, the findings suggest that similar levels of adherence may have been achieved through different pathways involving capability, opportunity, and motivation.

**Table 5:** Joint display of the mixed methods result.

Quantitative results and qualitative explanation		COM-B^a^ component
Early attrition		
	Illness/physical issues	Capability
	It is not for me	Motivation and opportunity
Similar adherence rate		
	Social support created in both groups	Opportunity
	Shared intervention components (ie, activity calendar, paper manual)	Opportunity, motivation, and capability
	Routine/habit formation	Motivation
	Enjoyment and reward	Motivation

^a^COM-B: Capability, Opportunity, Motivation-Behavior.

## Discussion

### Principal Findings

This pragmatic mixed methods pilot study evaluated the feasibility of a 26-week PA intervention delivered through online or on-site formats among older adults. Findings indicate that both delivery modes could be feasibly implemented in community settings and provide valuable insights into attrition, adherence, and participant experiences. Although no statistically significant differences were observed between delivery modes, the study was not designed or powered to assess comparative effectiveness. Rather, the findings should be interpreted as exploratory and hypothesis-generating, guiding future trials.

Attrition was a key feasibility indicator. The observed attrition rate (13/35, 37%) is comparable to those reported in previous exercise interventions among older adults [8,9], suggesting that long-term engagement remains challenging irrespective of delivery mode. Most dropouts occurred within the first 2 weeks, regardless of delivery mode, highlighting early vulnerability to disengagement. This finding is consistent with evidence that dropout often occurs early in programs [8,10]. Future trials may benefit from incorporating additional support during the initial stages of participation to reduce early withdrawal.

Adherence was used as a key indicator of intervention acceptability and participant engagement. Although adherence did not differ statistically between delivery modes, participants in both groups remained engaged throughout the intervention. Adherence in the online mode was comparable to levels reported in previous exercise interventions among older adults (eg, 55%-77%) [7], whereas adherence in the on-site mode was somewhat lower. Given the pilot design and limited sample size, these observed differences should be interpreted cautiously. Prior studies have reported differences between web- and paper-based interventions [26]; taken together with our data, this suggests a hypothesis for future research. Overall, our findings support the feasibility of delivering the intervention in both formats and highlight adherence as an important feasibility outcome in future trials.

Exploratory analyses suggested potential improvements in PA (GSLTEQ) and lower-body strength (ie, chair stand performance) over time, providing preliminary estimates and patterns for future trials rather than evidence of intervention effectiveness. Notably, the observed gains in chair stand performance are consistent with previous evidence supporting the OEP’s benefits for strength and physical function in older adults across different delivery formats [37,57].

Qualitative interviews provided explanatory insights into aspects influencing attrition and adherence. Attrition was related to physical issues (eg, pain, fatigue, chronic conditions, or acute illness/hospitalization limiting the ability to participate despite willingness) and a perceived lack of program fit (it’s not for me). By contrast, adherence was supported by 5 categories: appeal and reward (eg, perceived meaningfulness, enjoyment, mastery, safety/comfort, and experienced or anticipated health benefits); routine and commitment (eg, creating structure and habits that supported continuation); flexibility (eg, choice of timing, location, format, and equipment); feedback and PA overview (ie, self-monitoring via activity calendars for reflection and as a memory cue); and presence of others (ie, social belonging and accountability).

Mixed methods findings highlighted that capability-related constraints (eg, illness, pain, and fatigue) were the main reasons for attrition [8,9,12,14-16,58], especially early in the program [8,10]. Motivation and opportunity factors also contributed, as reflected in the qualitative finding “it’s not for me,” which aligns with prior research linking loss of interest [12] and preference mismatches to attrition [16]. Our findings suggest that better alignment between participants’ capabilities, preferences, identities, and social contexts may reduce attrition in future studies.

Mixed methods findings suggested an overlap in participant experiences across delivery modes. Although the online delivery mode was designed to increase physical opportunity through flexible, video-based access and the on-site delivery mode to enhance social opportunity through group-based training, participants frequently adapted their participation to their own circumstances. Many online participants created social contexts independently (eg, exercising with partners, friends, or neighbors), thereby incorporating elements of social support and accountability that had been intended to distinguish the on-site delivery mode. This highlights the value of social support for sustained participation [20,59,60], particularly when group members share similar social backgrounds, which may facilitate social interaction and adherence [13]. Similarly, participants in both delivery modes valued the shared intervention components (ie, paper manual and activity calendar). Participants reported that the activity calendar supported planning and self-monitoring [13,20] and that the paper manual supported engagement [59,62] and flexibility. Older adults commonly use external memory aids to remember action plans, which may support opportunity and adherence [63]. Motivational factors were particularly prominent in both delivery modes. Participants described both reflective processes (eg, understanding the purpose of exercise, valuing future health benefits, and establishing routines) and automatic processes (eg, enjoyment, emotional responses, and habit formation) as key drivers of adherence, consistent with previous findings that declining interest contributes to attrition [12].

Interpreted through the COM-B framework [19], attrition and adherence appear to depend on the interaction of capability, opportunity, and motivation. Capability factors, both physical and psychological, acted as both barriers (eg, physical limitations and reduced confidence due to a lack of personalized feedback) and facilitators (eg, self-monitoring). Opportunity factors, both social and physical, such as social support, flexibility, and varied exercise options, facilitated adherence, whereas poor social fit or program mismatch contributed to attrition. Motivation remained central, with both reflective (eg, future benefits and goal pursuit) and automatic (eg, enjoyment, habit formation, and emotional responses to feedback) processes supporting adherence. Taken together, these findings suggest that future interventions should prioritize support across all COM-B domains rather than focusing solely on delivery mode. They also suggest that similarities in intervention components may have influenced adherence more than delivery mode itself. Differences between the online and on-site delivery modes may have been less pronounced in practice than intended and may also have been obscured by the small sample size of this pragmatic pilot. The similar adherence patterns observed across delivery modes may therefore reflect this overlap in participant experiences.

From a feasibility perspective, both formats appear acceptable and generated meaningful participant experiences. Future studies should include larger samples and clearer differentiation between delivery modes if comparative effectiveness is to be evaluated. Future interventions should also support all COM-B domains by supporting capability during health fluctuations, providing flexible and accessible opportunities, and sustaining reflective and automatic motivation through reward, routine, feedback, and optional social connection. In other words, interventions should provide a variety of possibilities for PA to accommodate a heterogeneous and changing population [13,14]. Overall, our findings reinforce that older adults are a heterogeneous group with diverse capabilities and preferences for PA, suggesting that delivery mode alone is unlikely to determine attrition or adherence [13,14,20,61,64].

### Implications for Future Trial

Recruitment was substantially lower than planned. Future studies may benefit from broader recruitment strategies, additional sites, potentially longer recruitment periods, and more clusters to reduce the risk of site confounding and ensure adequate statistical power. Overall, future studies should test delivery strategies that preserve flexibility and social support while adding early-phase tailoring and maintenance supports to reduce early attrition and strengthen long-term behavioral adherence (ie, maintenance). To facilitate group comparisons between delivery modes, future trials could compare live online group sessions delivered by community hosts with on-site group-based sessions delivered by community hosts, thereby creating greater equivalence in social interaction across formats.

### Strengths and Limitations

A key strength is the pragmatic pilot-trial approach combined with embedded qualitative interviews, which improves ecological validity and enables interpretation of adherence and attrition processes in real-life contexts [43,65,66]. As expected for a pilot feasibility trial, the achieved sample size was insufficient to reliably detect modest between-group differences, thereby increasing the likelihood that true differences could remain statistically nonsignificant. Therefore, the absence of statistically significant between-group differences should not be interpreted as evidence of equivalence. The small achieved sample size also limits the generalizability of the findings. Recruiting participants was challenging, and future studies may benefit from incorporating older adults’ opinions and needs into intervention design through a mixed methods exploratory sequential design [31]. Future studies should also include a larger number of sites (ie, clusters) to facilitate participant recruitment and increase the sample size. Site-level allocation (clustered by residential area) also restricted randomization at the individual level and likely increased the likelihood that contextual influences (eg, community host practices and informal social support) would blur differences between delivery modes. Thus, the study design confounds delivery mode with site-specific factors, including community context and community host practices. While this was a reasonable design choice in the context of this study, future studies should consider individual-level randomization or include more sites. Our findings indicate promising acceptability of a simple, flexible, low-resource online intervention approach. However, the online delivery mode represented a low-support, asynchronous intervention rather than a digitally facilitated group intervention. As such, findings should not be generalized to interactive online exercise programs that include live sessions with peer interaction and coaching. To enhance comparability between delivery modes, future studies could ensure that interventions differ only in the mode of delivery (online vs on-site). For example, the online format could incorporate live sessions led by community hosts, mirroring the facilitated sessions provided in the on-site setting. Finally, measurement constraints (eg, relatively high baseline health, low depressive symptoms, and reliance on self-reported PA) may have reduced sensitivity to detect change in some outcomes.

### Conclusions

This pragmatic mixed methods pilot feasibility study suggests that both online and on-site delivery of a PA intervention are feasible and acceptable for many community-dwelling older adults. Patterns of attrition and adherence, together with participant experiences, provided insights into factors shaping engagement and highlighted the importance of supporting capability, opportunity, and motivation throughout the intervention. Attrition appeared to be related to physical health issues and perceived lack of fit, whereas adherence was facilitated by a combination of intervention appeal and perceived reward, routine formation and commitment, flexibility in formats and tools, feedback and PA overview through self-monitoring to support awareness and memory, and, often, the presence of others.

No statistically significant differences were observed between delivery modes; however, the study was not designed or powered to evaluate comparative effectiveness, and these findings should therefore be interpreted cautiously. Delivery mode itself appeared less influential than the extent to which the interventions support capability, opportunity, and motivation. These findings provide a rationale for future definitive trials to strengthen early-phase support and accommodate the diverse needs and preferences of older adults, for example, by offering a range of methods and opportunities to reengage following temporary illness. Larger trials are also warranted to evaluate intervention effectiveness, further compare delivery formats, and identify subgroups that may benefit most from specific delivery formats.

## Appendix

Multimedia Appendix 1 CONSORT 2010 checklist.

## Abbreviations

BF_01_: Bayes factor of H0 relative to H1
COM-B: Capability, Opportunity, Motivation-Behavior
CONSORT: Consolidated Standards of Reporting Trials
GDS: Geriatric Depression Scale
GSLTEQ: Godin-Shephard Leisure-Time Exercise Questionnaire
OEP: Otago Exercise Program
PA: physical activity
SES: socioeconomic status

## Data Availability

The datasets used and analyzed during this study are available from the corresponding author (JG) upon reasonable request.
